# Autaptic cultures of human induced neurons as a versatile platform for studying synaptic function and neuronal morphology

**DOI:** 10.1038/s41598-019-41259-1

**Published:** 2019-03-20

**Authors:** Pascal Fenske, M. Katharina Grauel, Marisa M. Brockmann, Anja L. Dorrn, Thorsten Trimbuch, Christian Rosenmund

**Affiliations:** 1 Institute of Neurophysiology, Charité – Universitätsmedizin, 10117 Berlin, Germany; 2 NeuroCure Cluster of Excellence, Charité – Universitätsmedizin, 10117 Berlin, Germany; 3 Berlin Institute of Health, Anna-Louise-Karsch-Straße 2, 10178 Berlin, Germany

## Abstract

Recently developed technology to differentiate induced pluripotent stem cells (iPSCs) into human induced neurons (iNs) provides an exciting opportunity to study the function of human neurons. However, functional characterisations of iNs have been hampered by the reliance on mass culturing protocols which do not allow assessment of synaptic release characteristics and neuronal morphology at the individual cell level with quantitative precision. Here, we have developed for the first time a protocol to generate autaptic cultures of iPSC-derived iNs. We show that our method efficiently generates mature, autaptic iNs with robust spontaneous and action potential-driven synaptic transmission. The synaptic responses are sensitive to modulation by metabotropic receptor agonists as well as potentiation by acute phorbol ester application. Finally, we demonstrate loss of evoked and spontaneous release by Unc13A knockdown. This culture system provides a versatile platform allowing for quantitative and integrative assessment of morphophysiological and molecular parameters underlying human synaptic transmission.

## Introduction

Recent technological advances provide access to human neurons through induction of iPSCs^1–5^ or direct conversion of fibroblasts into neurons^6,7^. This enables researchers to model human neurological diseases using patient-derived neurons or to study protein function in human neurons with a controlled genetic background^8,9^. So far, all studies investigating evoked neurotransmitter (NT) release in human induced neurons (hiNs) have been performed in mass culture^3,5,8–10^. However, their depth of analysis of synaptic properties and parameters was limited due to the network formed in this conventional culture system. Most studies therefore analysed only passive membrane properties, action potential (AP) generation and spontaneous activity within the network. Additionally, in some studies synaptic responses were evoked by extracellular or optogenetic stimulation of presynaptic neurons, but none of these techniques give a quantitative measure of the synaptic input and output of individual neurons or synaptic parameters, such as the vesicular release probability (P_VR_).

Autaptic cultures, in which single neurons grow in isolation on astrocytic microislands and form synapses exclusively with themselves^11^, provide an experimental system that allows the quantitative assessment of input and output properties of individual neurons, both in morphological and in functional experiments^12^. In addition, parameters such as vesicle fusogenicity, P_VR_, short-term plasticity and synaptic vesicle (SV) pool sizes can be assessed^13,14^. The autaptic culture system has proven particularly valuable in the analysis of presynaptic release mechanisms in rodent neurons, including docking/priming and the functionality of the fusion machinery^15–23^ and developmental phenotypes^12^. However, utilizing the standard protocol of murine autaptic neurons in our hands (Data not shown) as well as in others^24^ limited the accessibility of hiN autaptic cultures to experimental assessments, due to their long differentiation time and the poor survival rate of solitary hiNs.

Here, we report the first reliable method for autaptic cultures of hiNs. The two-phase protocol yielded single hiNs that reliably survived and matured to form fully functional synapses. The autaptic iNs displayed robust spontaneous and evoked NT release, which was modulated by metabotropic receptor agonists and potentiated by acute phorbol ester application. Finally, we demonstrated that this preparation is suitable for molecular interference as knocking down Unc13A using a shRNA-based approach was sufficient to abolish both spontaneous and evoked NT release. Taken together, the autaptic culture system for hiNs can serve as a reliable platform for studying human synaptic proteins and testing the genetics of human neuronal function.

## Results

### Establishment of an autaptic culturing protocol for hiNs

In initial experiments, we differentiated iPSCs into hiNs using the protocol described by Zhang and colleagues^5^ and plated the hiNs onto mouse-derived astrocytic microislands on day 4 post induction (DPI). We noticed that this approach resulted in low survival rates after 6 weeks in culture, with only a small fraction of hiNs suitable for electrophysiological assessment. These observations led us to hypothesize that hiNs require a period of significant cell-to-cell contact with other neurons in order to mature.

**Figure 1:**
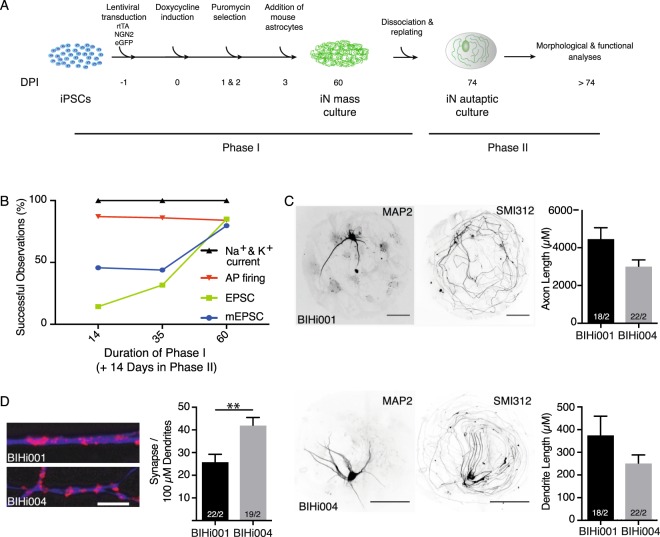
Human neurons in autaptic culture. (**A)** Schematic diagram of the autaptic culture protocol for hiNs. IPSCs were plated and infected with Ngn2, rtTA and eGFP on day −1, followed by application of doxycycline to start Ngn2 expression and puromycin selection on day 0 to 3. HiNs were co-cultured with mouse astrocytes for 60 days, followed by dissociation with Accutase and replating onto astrocytic microislands. Single autaptic iNs were analysed after 14–21 days. (**B)** Quantification of electrophysiological recordings of autaptic iNs replated on microislands after 14, 35 or 60 days post induction (DPI) in the mass-culture phase, followed by another 14–21 days of cultivation on microislands. (**C)** Representative images and quantification of total axonal and dendritic lengths of human autaptic iNs. Dendrites were identified by MAP-2 staining and axonal processes were stained with SMI-312. Images in each row belong to the same iN lines. Scale bars: 50 µm. (**D)** Example images and quantification of synapse density in human autaptic iNs. Synapses were identified by Synaptophysin labelling. Only synaptophysin punctae (red) on top of MAP-2 staining (blue) were counted. Scale bars: 5 µm. The numbers of neurons and independent cultures analysed are shown within the bars. Data are expressed as mean ± SEM.

We therefore adapted the differentiation protocol by splitting it into two phases: in the first phase we induced hiNs from iPSCs by forced expression of Ngn2 as described (Zhang *et al*., 2013) and allowed them to develop as mass culture. In the second phase we carefully dissociated the iNs and reseeded them onto astrocytic microislands (Fig. 1A,C). Despite the fact that hiNs grew extensive neurites during the mass-culture phase, we were able to efficiently dissociate them for replating. Replated iNs were subsequently cultured for 14–21 days before they were further analysed. Interestingly, the survival rate of iNs after replating was surprisingly high, only requiring a seeding density of 555 iNs/cm^2^ compared to 333 neurons/cm^2^ for murine hippocampal neurons derived from newborn animals^12^.

In order to find the optimal time point for replating the hiNs, we performed a timeline experiment, in which we replated hiNs at DPI 14, 35 and 60. We then used whole-cell patch-clamp recordings in order to examine the passive and active membrane properties of the autaptic hiNs after 14 to 21 days in autaptic culture (Fig. 1B). All autaptic iNs possessed voltage-dependent sodium and potassium currents and demonstrated robust AP firing as a function of injected current, regardless of the length of the mass-culture phase. In contrast only 44% of the autaptic iNs generated at DPI 35 exhibited spontaneous and 32% showed evoked NT release. In the autaptic iNs generated at DPI 14 this fraction was even less, with only 14.3% of the iNs having evoked postsynaptic responses. In the subsequent characterization we therefore exclusively considered human autaptic iNs replated at DPI 60 as 80% of the resulting autaptic iNs showed spontaneous and evoked NT release after 14–21 days (Fig. 1B).

The properties of iPSC lines generated by different methodologies can vary extensively^25^. For the following experiments we therefore generated autaptic hiNs from two independent iPSC lines available from the Berlin Institute of Health Core Facility Stem Cells (Berlin, Germany) to ensure that our protocol is generally applicable to iPSCs: the male iPSC line BIHi001-A derived from human foreskin fibroblasts using Sendai virus vectors and the female iPSC line BIHi004-A derived from normal human fibroblasts using episomal vectors.

### Morphology of hiNs in autaptic cultures

Using immunocytochemistry, we analysed morphological features of the DPI 60 + 14 autaptic iNs. They possessed the general features of differentiated neurons in culture, namely a compact cell soma, MAP2-positive dendritic trees and extensive neurofilament-positive axons. Quantitative analysis of MAP2-positive neurites showed that dendritic trees were rather short, with mean lengths of 377 ± 83 µm and 253 ± 35 µm in BIHi001- and BIHi004-derived iNs, respectively (Fig. 1C), and axonal outgrowth, exemplified by SMI312 labelling, was longer, reaching 4.5 ± 0.6 mm and 3.0 ± 0.3 mm in BIHi001- and BIHi004-derived iNs, respectively, than murine hippocampal neurons (Fig. 1C)^12,26^.

We quantified synapse formation by counting synaptophysin-positive punctae colocalising with MAP2-positive dendrites (Fig. 1D). Synapse densities were 26 ± 3 and 42 ± 3 per 100 µm in BIHi001 and BIHi004 iNs resulting in a total synapse number of 98 and 106 synapses per cell, respectively.

### Ion channel characteristics

**Figure 2:**
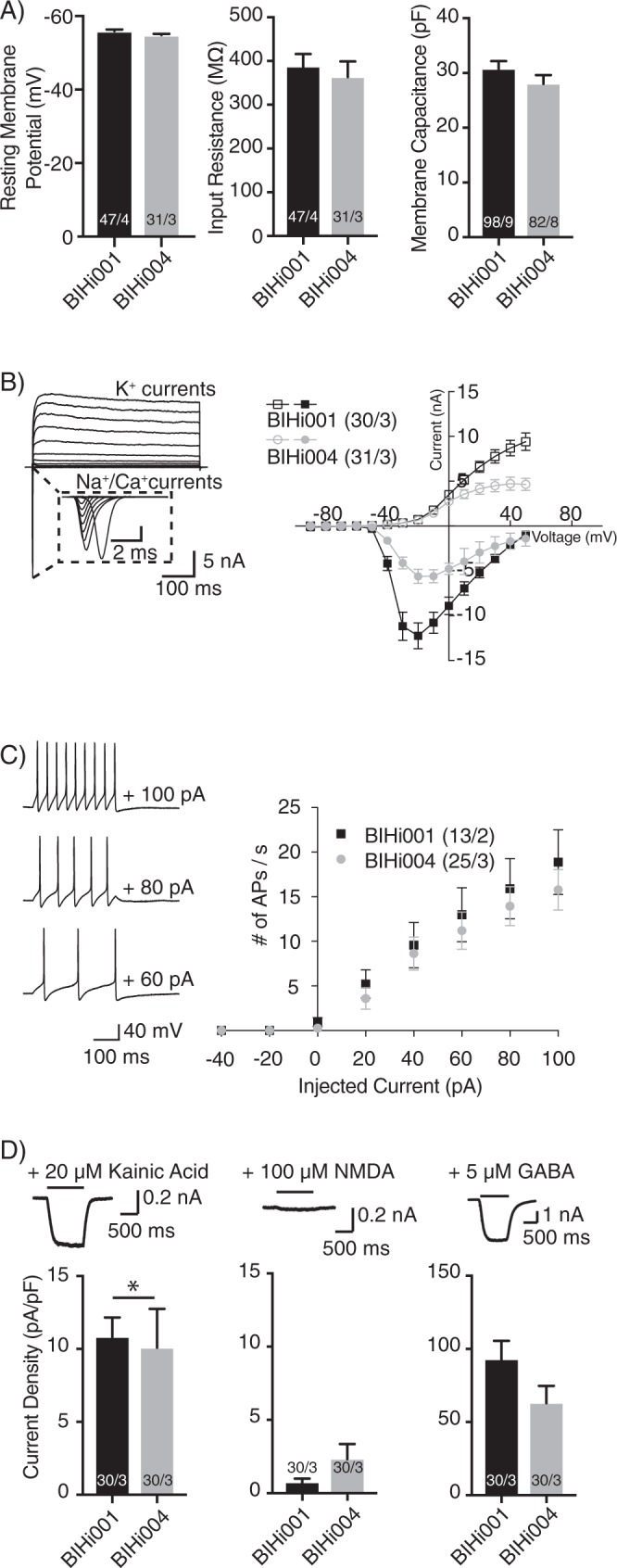
Passive membrane properties and ion channel characteristics of iNs in autaptic culture. (**A)** Average resting membrane potential, input resistance and membrane capacitance of autaptic iNs. (**B)** Representative traces (left) and quantitative analysis of whole-cell voltage-clamp Na^+^/Ca^2+^ and K^+^-currents (right). (**C)** Representative traces (left) and quantitative analysis of the number of APs per second elicited by current injections in current-clamp recordings (right). (**D)** Quantification of whole-cell currents evoked by application of either 20 µM kainic acid (left), 100 µM NMDA (middle) or 5 µM GABA (right) in the presence of 10 µM glycine and in the absence of Mg^2+^. The numbers of neurons and independent cultures analysed are shown within the bars. Data are expressed as mean ± SEM.

**Figure 3:**
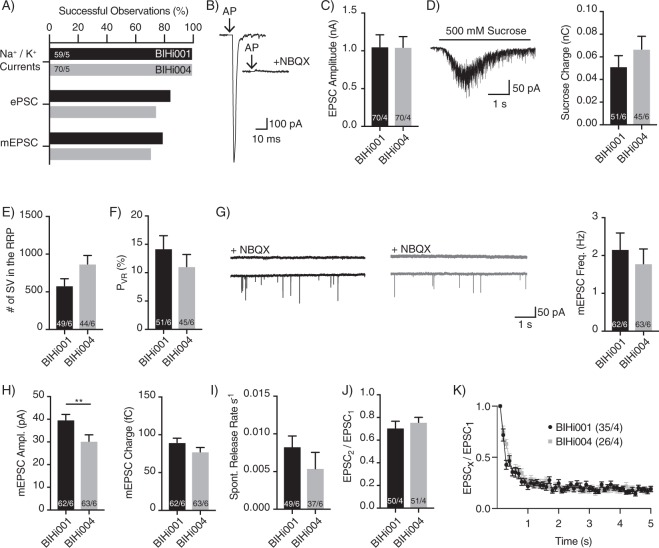
AP-evoked and spontaneous NT release and vesicular release probability of autaptic iNs. (**A)** Fraction of neurons which showed membrane voltage-dependent K^+^ and Na^+^/Ca^2+^ currents, EPSC and mEPSC in autaptic hiN cells. (**B)** Representative traces of AP-evoked EPSCs recorded in the absence or presence of the AMPA receptor antagonist NBQX. (**C**) Mean AP-evoked EPSC amplitudes of iNs induced from BIHi001- and BIHi004-iPSCs. (**D**) Exemplary trace of the synaptic response of an autaptic iN elicited by 500 mM hypertonic sucrose solution (left) and average size of the RRP charges (right). (**E,F)** Number of SVs in the RRP (**E**) and mean P_VR_ (**F**). (**G)** Exemplary mEPSCs recorded from autaptic iNs in the absence or presence of NBQX (left) and mean mEPSC frequencies in BIHi001- and BIHi004-derived iNs (right). (**H)** Mean amplitudes and charges of mEPSCs. (**I)** Spontaneous release rate of human autaptic iNs. (**J)** Average paired-pulse ratios (PPR) calculated from two EPSCs with an ISI of 25 ms. (**K)** Normalized EPSC amplitudes during a 10 Hz train stimulation. AP artefacts were blanked and are indicated by black arrows. The numbers of neurons and independent cultures analysed are shown within the bars. Data are expressed as mean ± SEM.

We used whole-cell patch-clamp recordings in order to examine the passive and active membrane properties of the autaptic human iNs (Fig. 2) to validate their maturity. Neurons derived from BIHi001- or BIHi004-iPSCs had a similar mean resting membrane potential of −55 mV, input resistance of approximately 375 MΩ and membrane capacitance around 30 pF (Fig. 2A). All autaptic iNs possessed voltage-dependent sodium and potassium currents (Fig. 2B, also see Fig. 3A). Current-clamp experiments also demonstrated robust action potential firing as a function of injected current (Fig. 2C).

We quantified excitatory and inhibitory NT receptor expression in the hiNs by exogenous application of the respective agonists (Fig. 2D). Application of kainic acid (20 µM) elicited a mean current density of approximately 10 pA/pF (Fig. 2D, left), indicating AMPA-type glutamate receptor expression. In contrast, application of NMDA (100 µM) yielded negligible currents, indicating poor expression of NMDA receptors (Fig. 2D, middle). Application of GABA (5 µM) led to robust responses in all iNs tested (Fig. 2D, right). These results indicate expression of GABA receptors in iNs from both iPSC lines (BIHi001-, 92.9 ± 12.6 pA/pF and BIHi004-derived hiNs, 67.02 ± 12.37 pA/pF).

### Neurotransmission in autaptic hiNs

Upon AP induction, a large majority of the cells (85%) responded with a synaptic inward current, demonstrating the high efficiency of our protocol for generating functional, mature, autaptic hiNs (Fig. 3A). We did notice that the time between induction of APs and postsynaptic responses was more variable compared to murine autaptic cultures^11^, which we attributed to more extensive axonal outgrowth in the hiNs (Fig. 1C). The postsynaptic responses were quantitatively blocked by saturating concentration of the AMPA receptor antagonist NBQX, confirming a glutamatergic phenotype (Fig. 3B). The responses were robust with mean amplitudes of 1.1 ± 0.2 nA and 1.0 ± 0.1 nA in BIHi001- and BIHi004-derived hiNs, respectively (Fig. 3B,C).

One of the major advantages of the autaptic culture system is that it allows for quantification of the readily-releasable pool (RRP) of vesicles, which is a product of the number of fusion-competent vesicles per synapse and the number of synapses. The RRP is determined by application of hypertonic sucrose solution onto the neurons using a fast-flow perfusion system^27^. This results in a transient inward current, which can be quantified by integration (Fig. 3D). The mean RRP charges were 50 ± 10 pC and 67 ± 11 pC in BIHi001- and BIHi004-derived hiNs, respectively (Fig. 3D).

In the absence of stimulation, we recorded spontaneous NT release indicated by miniature excitatory postsynaptic currents (mEPSCs) (Fig. 3G). The mean mEPSC amplitude was significantly smaller in BIHi004- compared to BIHi001-derived hiNs (Fig. 3H) (BIHi001-: 39.7 ± 2.4 pA; BIHi004-derived hiNs: 30.4 ± 2.8 pA; p = 0.0024), but spontaneous release frequencies were around 2 Hz for both hiN lines (Fig. 3G). We next quantified the mEPSC charge and divided it by the RRP charge to compute the total number of SVs in the RRP. BIHi001-derived hiNs had an average of 618 ± 97 vesicles (Fig. 3E), which results in an average of 6.2 fusion-competent vesicles per synapse. BIHi004-derived iNs had an average of 869 ± 113 vesicles, which equals 8.2 fusion-competent SVs per synapse.

Knowing the RRP and the EPSC charges we determined the fusion efficiency of individual fusion-competent vesicles by calculating the vesicular release probability (P_VR_). P_VR_ is the likelihood that a given fusion competent SV is released in response to an AP. We found that the P_VR_ was 14.2 ± 2.3% and 11.1 ± 2.1% in BIHi001- and BIHi004-derived iNs, respectively (Fig. 3F).

We also calculated the spontaneous release rates of individual fusion-competent vesicles by normalizing mEPSC frequency (Fig. 3G) to RRP size (Fig. 3E). HiNs had a high spontaneous release rate, where 0.8 ± 0.1% and 0.5 ± 0.2% of the RRP is turned over within a second in BIHi001- and BIHi004–derived hiNs, respectively (Fig. 3I).

The relatively high release probability and high spontaneous release rates in the hiNs suggest high vesicle fusogenicity^13^. This hypothesis was supported by the relatively strong depression of the EPSCs in response to a paired-pulse stimulus (Fig. 3J) as well as 10 Hz train stimulation (Fig. 3K) observed in both hiN lines.

### Postsynaptic responses are mediated by AMPA receptors in autaptic hiNs

**Figure 4:**
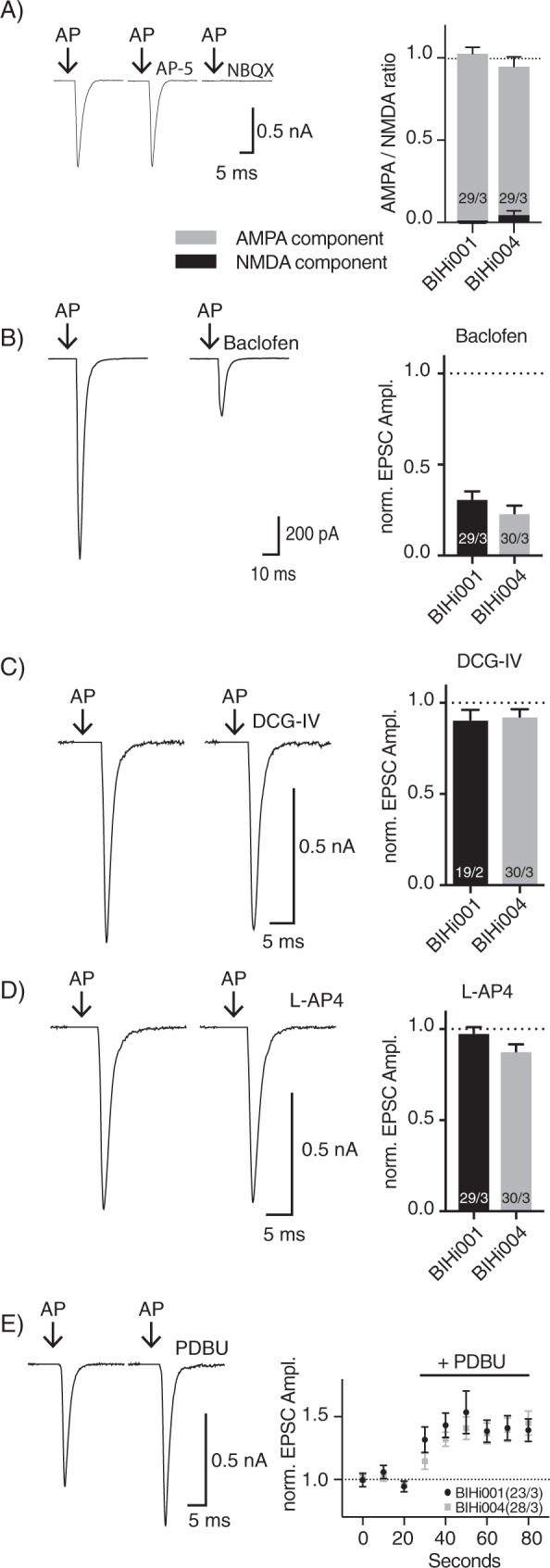
Modulation of glutamate release by metabotropic receptors and phorbol esters. (**A)** Components of the EPSCs mediated by AMPA and NMDA receptors. **(B–D)** Comparison of EPSC amplitudes with and without Baclofen (**B**), L-AP4 (**C**) and DCG-IV (**D**). (**E)** EPSC potentiation by application of PDBu. AP artefacts were blanked and are indicated by black arrows. The numbers of neurons and independent cultures analysed are shown within the bars. Data are expressed as mean ± SEM.

We utilized specific antagonists for AMPA (NBQX; 3 µM) and NMDA receptors (D-AP5; 10 µM) at saturating concentrations in order to examine the glutamatergic receptor composition at the hiN postsynapse. The results differed between hiN lines, with BIHi001-derived iNs showing no evidence of NMDA receptor - mediated synaptic transmission, while in BIHi004-derived iNs the NMDA receptors contributed around 5% to the EPSC amplitude (Fig. 4A). These data indicate a general deficiency in NMDA receptor expression or surface location, consistent with our results obtained from exogenous application of AMPA and NMDA receptor agonists (Fig. 2D) and with gene expression analysis performed by others^5^.

### Modulation of glutamate release by metabotropic receptors and phorbol esters

Release from human glutamatergic synapses is also modulated by well-known modifiers of release probability. First, the GABA_B_ agonist Baclofen (20 µM), which inhibits release from glutamatergic synapses^28^, rapidly and reversibly reduced EPSC amplitudes by 69% and 77% in BIHi001- and BIHi004- derived iNs, respectively (Fig. 4B). These results demonstrate that the human glutamatergic iNs generated by forced expression of Ngn2 express presynaptic GABA_B_ receptors and efficiently couple them to the release apparatus.

Second, release is also modulated by metabotropic glutamate receptors (mGluRs). 1 µM DCG-IV, the agonist for type II mGluRs, inhibited synaptic transmission significantly in both iN lines (9.2% in BIHi001-derived iNs; 7.4% in BIHi004-derived iNs; Fig. 4C). In contrast, only BIHi004-derived iNs responded to activation of type III mGluRs by the agonist L-AP4 (30 µM) with a small but significant reduction of EPSC amplitudes by 12% (Fig. 4D).

Finally, synaptic responses are potentiated by phorbol esters, which are known to modulate vesicle fusogenicity through activation of the Unc13 proteins^13,21^. Application of 1 µM of PDBu rapidly increased the evoked responses, plateauing at 123% and 142% in BIHi001- and BIHi004-derived iNs, respectively (Fig. 4E). The overall degree of potentiation was modest, consistent with the high initial release probability^13,29^ (Fig. 3).

### Knockdown of Unc13A abolishes evoked and spontaneous NT release

**Figure 5:**
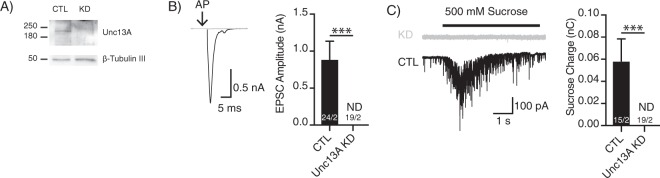
Loss of function by knockdown of Unc13A in BIHi001. (**A)** Immunoblot of Unc13A protein levels of iNs transduced with a shRNA against Unc13A or a control construct. Signal at 200 kDa corresponds to the expected Unc13A, and the signal at 50 kDa to β-Tubulin. Western blot images have been cropped for presentation. Full size image are presented in Supplementary Fig. S1. (**B)** Exemplary traces of AP-evoked EPSCs (left) and average EPSC amplitudes (right). (**C)** Exemplary traces of the currents evoked by 500 mM hypertonic sucrose solution (left) and average sizes of the RRP charges (right) in KD and control (CTL) conditions. (ND, not detectable). AP artefacts were blanked and are indicated by black arrows. The numbers of neurons and independent cultures analysed are shown within the bars. Data are expressed as mean ± SEM.

We developed the autaptic hiN protocol also to study human synaptic protein function in the human cellular background. To test whether it is sufficient to genetically modify the hiNs during replating, we developed a shRNA-based knockdown (KD) assay for Unc13A, a homologue of the murine priming factor Munc13-1. Expression of Unc13A-specific shRNA in iNs by means of lentiviral infection led to a nearly complete reduction of Unc13A protein level when tested in the mass-culture (Fig. 5A) and to a complete loss of evoked NT release in autaptic hiN cultures (Fig. 5B). Application of hypertonic sucrose solution showed that there was no measurable RRP in these hiNs (Fig. 5C). These results are consistent with an indispensable role of Unc13A in the priming of SVs, as it has been observed in murine loss-of-function models^23^.

## Discussion

Here, we established the first two-phase protocol for the autaptic culture of human iPSC-derived neurons. Using two independent iPSC lines we demonstrate that our method reliably and reproducibly generates mature hiNs growing in isolation on astrocytic microislands and forming functional synapses onto themselves. Additionally, we successfully modified gene expression in the autaptic hiN leading to major changes in the observed synaptic properties.

To establish autaptic cultures of hiNs we had to overcome two major difficulties: the limited life time of the astrocytic microislands^24^, and the fact that hiNs have a low survival rate when grown in isolation early on during their differentiation process. To circumvent both obstacles, we split the culturing protocol into two phases: in the first phase the hiNs were allowed to mature in a mass culture setting, after which they were replated onto astrocytic microislands for a second “autaptic” culturing phase. Timeline experiments indicated that the iNs indeed needed 60 days of cell-to-cell contact to properly mature. This time is not necessary to build synapses for functional autaptic connections as during replating the iNs lose the majority of their neurites and subsequently reform synapses during the two-weeks autaptic phase.

Overall, the autaptic iNs had passive membrane properties comparable to primary murine cultured neurons^30^ and mass cultured hiNs derived from embryonic stem cells^3,5,8–10,31^. While the overall cellular morphology also appeared mature, dendrites were relatively short when compared to primary mouse autaptic neurons^12,26^. We think this is not specific for hiNs but rather caused by the replating or culturing procedure because estimates for mass-cultured iN dendrites are longer^3,9,10^. In contrast, the axonal outgrowth appears much more resilient to replating, as axon lengths were comparable to estimates from mass-cultured hiNs^10^ and were 50–250% longer than those of primary mouse hippocampal neurons in autaptic culture^12^. The synapse densities were comparable to those of autaptic rodent neurons^12,26^, but because of the shortened dendritic lengths, the total synapse numbers were reduced accordingly.

The functional properties of the autaptic iNs were overall consistent with the mass-cultured iNs described by Zhang and colleagues, which also showed a lack of NMDA receptor expression^5^. However, the synaptic responses we recorded were much larger than those measured in mass culture^5,8,9,31^. Depending on the presynaptic stimulation technique (e.g. paired recordings, field stimulation) the responses in mass culture only reflect a small and highly variable fraction of the synaptic input onto the recorded postsynaptic neuron. In contrast, the autaptic culture system has the unique advantage that inputs are monosynaptic and originate all from the same neuron. Therefore, it allows for quantitative assessment of the total synaptic input and output of a given neuron with a single AP stimulation.

The combined functional and morphological analysis in the autaptic hiNs allowed us to describe in more detail the properties of individual human synapses. For example, we noticed that the number of readily-releasable SVs per synapse was in the range of 5 to 10 vesicles, a value similar to that reported in rodent hippocampal neurons^32,33^. Computation of release probability and spontaneous release rates indicated that hiN vesicles display rather high fusogenicity, reminiscent of high release probability glutamatergic neurons, such as layer 4 cortical neurons^34^. The mechanism for the rather high fusogenicity is unknown but may be related to the expression of the vesicular glutamate transporter subtype 2^5^, which is known to be associated with intrinsically higher release probability^35^. High vesicle fusogenicity is also consistent with the moderate potentiation by PDBu application^13,29^.

Modulation of NT release by activation of presynaptic metabotropic receptors and their associated second messenger systems plays an important role for proper neuronal and brain function^36^. Metabotropic receptors are targets in the drug development and treatment of several neuropsychiatric disorders^37–39^. The identification of the particular set of metabotropic receptors expressed by hiNs is therefore particularly important. At the same time, assessment of the modulation of NT release by second messenger systems requires reliable quantification of evoked synaptic responses, which is the major advantage of the autaptic cell culture system. Using our autaptic culture system for hiNs, we showed that synaptic transmission of these neurons is reliably modulated by GABA_B_ and metabotropic glutamate receptors.

While our analyses identified variations in some of the morphological and functional properties of the two iN lines, the overall differences were rather small, emphasizing the robustness and stability of our system. Those differences we encountered may have arisen from genetic and epigenetic variations within the iPSC lines^40^.

We demonstrated that using shRNA-based KD assays during replating is sufficient to generate *loss-of-function* models for synaptic proteins. The KD of Unc13A recapitulated the complete disruption of synaptic NT release in *Munc13-1/2* DKO neurons^23^. Hence, the here described two-phase protocol for autaptic iNs is especially flexible for protein structure-function experiments and modelling of human diseases, because it is sufficient to keep one or two lines of wildtype iNs during the mass-culture phase, which can then be used to study different protein functions during the autaptic culturing phase.

Our autaptic culture technique constitutes a versatile platform for multilevel phenotypic analyses of hiNs. With the here described technique, alterations in important synaptic parameters, such as the number of fusion-competent vesicles and their individual release probability, can now be more comprehensibly analysed and compared in a much more quantitative manner than with mass culture techniques. In conjunction with *in situ* genome editing, it will allow the identification and analyses of developmental, morphological and/or functional aspects of proteins of interest. The electrophysiological, pharmacological and immunocytochemical analyses of neuronal morphology and function shown here can be extended to a two-neuron microcircuit system to study the interaction of different cell types or mutant and wildtype cells^12,26,41,42^. The autaptic culture system also represents a powerful tool to distinguish between cell-autonomous and non-cell-autonomous regulatory processes^12,26^ and to describe the cellular phenotypes associated with neurological disorders such as Rett syndrome^12,43,44^, and neurodegenerative diseases, such as Alzheimer’s Disease^45,46^. In addition the autaptic cell culture technique can also be combined with other common methods, such as calcium indicators and vesicle pH dynamics imaging^47^ or electron microscopy that would allow for further analyses of calcium signalling and the SV cycle. Additionally, combining the electrophysiological functional analysis with single cell RNA sequencing in a Patch-Seq approach^25,48^ will aid in identifying molecular signalling pathways underlying a particular cellular phenotype.

## Experimental Procedures

### Ethical statement

All animals used were handled in accordance with the relevant guidelines and regulations. Protocols were approved by the ‘Landesamt für Gesundheit und Soziales’ (LaGeSo; Regional Office for Health and Social Affairs) in Berlin and animals reported under the permit number T0220/09.

### Virus Generation

Lentiviruses were produced by cotransfection of HEK293T cells with 10 µg envelope/packaging plasmids (3,9 µg pRSV-REV (Addgene Plasmid #12253), 8,1 µg pMDLg/pRRE (Addgene Plasmid #12251), 6 µg VSVg (Addgene Plasmid #12259)) and 12 µg cDNA transfer plasmids per 75 cm^2^ culture area using PEI MAX (Polyscience) transfection^49^. Supernatants containing lentiviruses were harvested 48 hr after transfection and purified using Amicon Ultra-15 Centrifugal Filter units Ultracel-100 K (Merck Millipore). Only virus batches with >90% infection efficiency, as assessed by fluorescent reporter expression or puromycin resistance, were used for experiments. cDNA constructs for rtTA (FUW-rtTA), Ngn2 (FUW-TetO-Ngn2-T2A-Puro) and eGFP (FUW-eGFP) expression were kindly provided by Prof. Thomas C. Südhof. For Unc13A KD a synthetic shRNA oligonucleotides (sequence: 5′ ggacaccatcaagcaatat ttcaagaga atattgcttgatggtgtcc ttttttccaa 3′) was inserted into a FUGW shuttle vector^50^ containing a U6 promoter and a synapsin promoter driven NLS-RFP reporter gene.

### Generation of hiN Cells from human iPSCs

IPSCs were obtained from two independent iPSC lines provided by the Berlin Institute of Health Core Facility Stem Cells (Germany): The BIHi001-A (https://hpscreg.eu/cell-line/BIHi001-A) and BIHi004-A (https://hpscreg.eu/cell-line/BIHi004-A) iPSC lines. IPSCs were maintained as feeder-free cells in Essential 8 flex medium (ThermoFisher Scientific) in a standard incubator (37 °C, 5% CO_2_).

Excitatory hiNs were produced as described previously^5^. In brief, iPSCs were dissociated with Accutase cell dissociation reagent (ThermoFisher Scientific), plated on Matrigel (Corning) - coated 6-well plates (1,7 × 10^5^ cells/cm^2^) in Essential 8 flex medium supplemented with 2 µM thiazovivin (Tocris) and infected with lentiviral vectors on day −1. On day 0, the medium was changed to DMEM/F12 (ThermoFisher Scientific) containing N-2 supplement (ThermoFisher Scientific), non-essential amino acids (ThermoFisher Scientific), human BDNF (10 mg/l, PeproTech), human NT-3 (10 mg/l, PeproTech), mouse laminin (0.2 mg/l, ThermoFisher Scientific) and doxycycline (2 µg/ml, Sigma Aldrich) to induce TetO-dependent gene expression. Doxycycline was retained in the medium until the end of the experiments. On day 2 and 3 DMEM/F12 medium was replaced and 0.5 mg/l puromycin was included to select only induced iNs. On day 4 medium was changed to Neurobasal-A medium (ThermoFisher Scientific) containing B-27 supplement (ThermoFisher Scientific), GlutaMAX (ThermoFisher Scientific) human BDNF (10 mg/l), human NT-3 (10 mg/l), mouse laminin (0.2 mg/l), Cytosine β-D-arabinofuranoside (Ara-C) (2 mg/ml, Sigma Aldrich) and doxycycline (2 µg/ml) and mouse glial cells were included (3 × 10^5^ cells/cm^2^). From day 7 on half of the medium in each well was replaced every 5 days with Neurobasal-A medium containing B-27 supplement, GlutaMAX, doxycycline (2 µg/ml) and 2.5% fetal bovine serum (PanBiotech). After 50–70 days, cells were washed twice with 0.5 mM EDTA/PBS (ThermoFisher Scientific) and dissociated with Accutase (2 x for 7 min at 37 °C). GFP-positive cells were seeded in a density of 3.3 × 10^3^ cells/cm^2^ onto astrocyte microislands to obtain autaptic cultures. Astrocytic islands were produced from mouse cortical glial cells derived from newborn C57/Bl6 mice. Microislands of growth-permissive substrate (collagen I (ThermoFisher Scientific), and poly-D-lysine (Sigma Aldrich, St. Louis, MO, USA)) were printed onto 30 mm round coverslips precoated with agarose type 4 (ThermoFisher Scientific). Astrocytes were plated at a density of 5 × 10^3^ cells per cm^2^ onto the microdot-coated coverslips one week before iNs were added. These cultures were maintained for another 13–21 days in a humidified incubator (37 °C and 5% CO_2_).

### Immunocytochemistry

Cells were fixed between day 80 and 121 in prewarmed 4% paraformaldehyde (PFA, Sigma Aldrich)/4% sucrose (Sigma Aldrich) in PBS (Merck Millipore) for 10 min at room temperature (RT). Cells were permeabilized for 15 min in PBS/0.1% Tween-20 (Carl-Roth GmbH + Co. KG), incubated in quenching solution (100 mM glycine in PBS) for 30 min and blocked in 5% normal donkey serum (Jackson ImmunoResearch Inc.) for 1 hour at RT. Cells were incubated with primary antibodies over night at 4 °C. The following primary antibodies were used: chicken anti-microtubule-associated protein 2 (MAP2) (1:2000; Merck Millipore, AB5543), mouse anti-pan-axonal neurofilaments (SMI-312; 1:1000; Covance Inc.); mouse anti-synaptophysin (1:200; Synaptic Systems). After washing the cells 3 x with PBS-T, primary antibodies were labelled with secondary antibodies Alexa-Fluor 405 or 647 (1:500; Jackson ImmunoResearch Inc.) for 1 hr at RT. Cells were washed 2 x in PBS-T and once in PBS and mounted on glass slides with Mowiol (ThermoFisher Scientific).

### Western blot

For quantification of Unc13A protein levels, hiN cells were lysed 14 days post transduction at 4 °C with lysis buffer (50 mM Tris, pH 8.0, 150 mM NaCl, 0.2% NP-40, protease inhibitor cocktail complete mini (Roche Diagnostics)). Equal amounts of total protein from the lysates of Unc13A-KD or scrambled control virus were separated on SDS polyacrylamide gel and transferred to a nitrocellulose membrane. Membranes were blocked for 1 hour with 5% skim milk in PBS-T and incubated at 4 °C over night with primary antibodies: anti-Munc 13-1 (126103 Synaptic Systems) and anti- β-Tubulin III (T8660 Sigma-Aldrich). Secondary antibodies were horseradish peroxidase-conjugated (Jackson ImmunoResearch). Detection was performed by using ECL Plus Western Blotting Detection Reagents (GE Healthcare Biosciences) in a Fusion FX7 detection system (Vilber Lourmat). Data were analysed offline using ImageJ.

### Morphological analysis

For morphological analysis, stacks of 16-bit fluorescence images were acquired on an Olympus IX81 inverted microscope equipped with a 10X or 20X objective (neurite length analysis) or a 60X water immersion objective (synaptic density analysis). Images were acquired with a CCD camera (Princeton MicroMax; Roper Scientific) using MetaMorph software (Molecular Devices). All images were analysed using ImageJ software (National Institutes of Health). For each image stack maximum intensity projection were generated. Uniform background subtraction and optimal threshold adjustment was performed on all images. Quantification of MAP2-positive or neurofilament (SMI312)-positive processes with the NeuronJ plugin was used to determine total dendritic and axonal lengths. The density of synaptophysin-positive punctae overlapping with MAP2-positive dendrites was determined using a custom-written ImageJ plugin. Two independent cultures were imaged and analysed per group for every experiment.

### Electrophysiology

Whole-cell patch-clamp recordings were performed between day 63 and 91 post neuronal induction at RT using a Multiclamp 700B amplifier (Molecular Devices). The series resistance was compensated by 70% and only cells with series resistances of <12 MΩ were analysed. Data were acquired at 10 kHz using pClamp 10 software (Molecular Devices) and filtered using a low-pass Bessel filter at 3 kHz. Data were analysed offline using Axograph X (Axograph Scientific). The patch pipette solution contained 136 mM KCl, 17.8 mM HEPES, 1 mM EGTA, 0.6 mM MgCl_2_, 4 mM ATP-Mg, 0.3 mM GTP-Na, 12 mM phosphocreatine, and 50 units/mL phosphocreatine kinase (300 mOsm, pH 7.4, all Carl-Roth). The recording chamber was constantly perfused with extracellular solution containing 140 mM NaCl, 2.4 mM KCl, 10 mM HEPES, 2 mM CaCl_2_, 4 mM MgCl_2_, and 10 mM glucose (pH adjusted to 7.3 with NaOH, 300 mOsm, all Carl-Roth). For experiments examining postsynaptic receptors Figs 2D and 4A we used a Mg^2+^-free extracellular solution containing 10 µM glycine. Solutions were applied using a fast-flow perfusion system.

For whole-cell voltage clamp recordings, neurons were maintained at −70 mV holding potential. Cells were subjected to 500 ms depolarizations in 10 mV steps from −90 mV to +50 mV to analyse Na^+^/Ca^2+^ and K^+^ currents.

AP firing patterns and passive membrane properties were recorded in whole-cell current-clamp configuration. Bridge balance was automatically compensated. 300 ms current steps from −40 pA to 120 pA were injected. The resting membrane potentials were analysed from baseline and the input resistances were calculated by Ohms law using a current step of −50 pA for 600 ms.

Membrane standing glutamatergic and GABAergic ionotropic receptor function was tested by pulsed application (1 s) of 20 µM kainic acid, 100 µM *N*-Methyl-D-aspartic acid (NMDA) and 5 µM γ-Aminobutyric acid (GABA) (all Tocris). Peak amplitudes from six consecutive sweeps were averaged. The values were normalized to the cell capacitance.

EPSCs were evoked by 2 ms somatic depolarization from −70 to 0 mV producing an unclamped axonal AP. To determine the size of the RRP, 500 mM sucrose (Sigma Aldrich) solution was applied for 5 s and the resulting transient inward current was integrated^27^. mEPSC were detected using a sliding template function in AxoGraph X^51^ and false positive events were corrected by using the selective competitive receptor antagonist 2,3-Dioxo-6-nitro-1,2,3,4-tetrahydrobenzo[f]quinoxaline-7-sulfonamide (NBQX; 3 µM (Tocris)). The number of vesicles in the RRP was calculated by dividing the charge of the sucrose response by the average mEPSC charge for each neuron. The P_VR_ was calculated as the ratio of the average EPSC charge and the RRP charge. The spontaneous release rate was determined by dividing the mEPSC frequency by the number of vesicles in the RRP for each cell. The paired-pulse ratio (PPR) was calculated by applying two stimuli with an inter-stimulus interval (ISI) of 25 ms and dividing the amplitude of the second EPSC by that of the first EPSC.

For pharmacological studies with 30 µM L-(+)-2-Amino-4-phosphonobutyric acid (L-AP4) (Tocris), 20 µM R-Baclofen (Tocris), 10 µM D-(−)-2-Amino-5-phosphonopentanoic acid (D-AP5) (Tocris), 3 µM NBQX (Tocris) and 1 µM (2S,2′R,3′R)-2-(2′,3′-Dicarboxycyclopropyl) glycine (DCG IV) (Tocris) baseline EPSC amplitudes were monitored at 0.2 Hz for 30 s without drug application, followed by 30 s in the presence of the drug and another 30 s without drug application. The degree of EPSC depression or potentiation was determined by dividing the mean EPSC amplitude in the presence of the drug by the mean EPSC amplitude before drug application in extracellular solution of the same cell. The degree of EPSC potentiation of 1 µM phorbol-12,13-dibutyrate (PDBu) (Tocris) was determined by monitoring EPSC amplitudes at 0.2 Hz for 15 s in standard extracellular solution followed by 30 s with drug application. The degree of potentiation was calculated by normalizing to the first EPSC amplitudes recorded in standard extracellular solution.

### Statistics

Data were collected from 2–9 independent hiN autaptic cultures (replicates N) to account for putative culture to culture variability. For each replicate, hiNs were generated by an individual induction process from each human iPSC line and these hiNs were then used only for one autaptic culture. For electrophysiological experiments, we obtained an equal number of recordings in the range of 5 to 15 cells per experimental group in each replicate culture. For details see Supplementary Table S1.

Statistical significance was determined by using the two-tailed Mann Whitney test for unpaired data at the given significance level (*p < 0.05, **p < 0.01, ***p < 0.001) using GraphPad Prism 7.

## Supplementary information

Supplementary File

## Data Availability

Data generated and analysed in this study are available from the corresponding author upon request.
