# Supplementary material for: Autaptic cultures of human induced neurons as a versatile platform for studying synaptic function and neuronal morphology
Source: Sci Rep. 2019 Mar 20;9:4890. doi: 10.1038/s41598-019-41259-1 (PMC6427022; doi:10.1038/s41598-019-41259-1)
Supplement: Supplementary file 1 — Supplementary File [file 41598_2019_41259_MOESM1_ESM.pdf]

Supplementary Information

Autaptic cultures of human induced neurons as a versatile platform for studying synaptic function and neuronal morphology

Pascal Fenske, M. Katharina Grauel, Marisa M. Brockmann, Anja L. Dorrn, Thorsten Trimbuch, and Christian Rosenmund

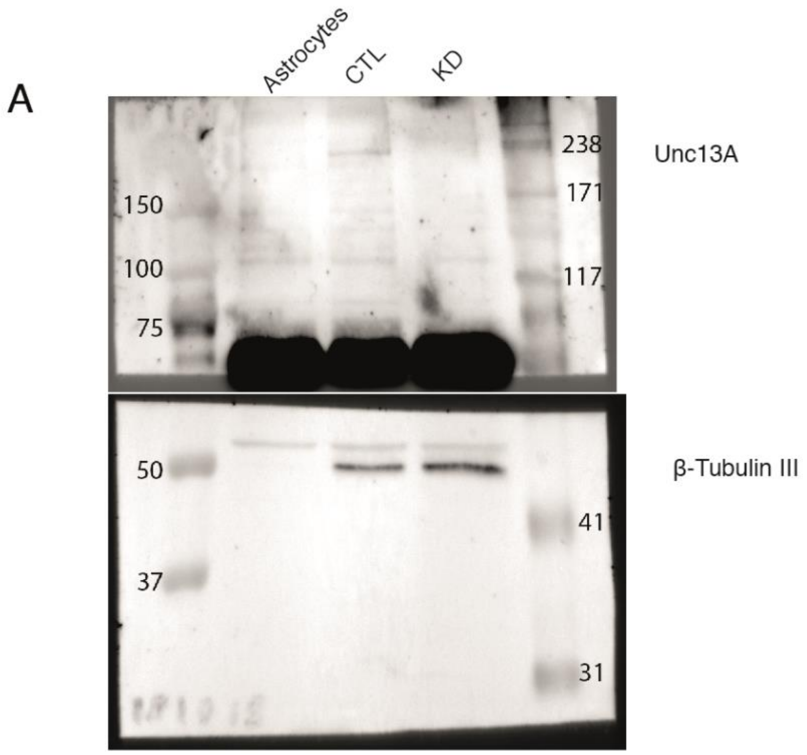

Supplementary Figure S1. Western blot of Unc13A knockdown
A) Full size immunoblot image of Unc13A protein levels of iNs transduced with a shRNA against Unc13A or a control construct, as well as a pure astrocyte culture. Signal at 200 kDa corresponds to the expected Unc13A, and the signal at 50 kDa to β-Tubulin. Signals in astrocyte loading control are unspecific binding sites of the used antibodies.

Supplementary Table S1. Detailed statistical data

Figure	Parameter measured	Statistical Test	Number of replicates (N)	Cellline	Cells per replicate	Cells in total (n)	Mean	SEM	p-value
1 C	Axon length	two-tailed Mann Whitney test	2	BIHi001	8-10	18	4480	580.3	0.054
				BIHi004	10-12	22	3025	335.5	
1 C	Dentrite length	two-tailed Mann Whitney test	2	BIHi001	8-10	18	376.7	82.92	0.4755
				BIHi004	10-12	22	252.7	35.48	
1 D	Synapse density	two-tailed Mann Whitney test	2	BIHi001	10-12	22	26	3.26	0.0034
				BIHi004	9-10	19	42.12	3.387	
2 A	Resting membrane potential	two-tailed Mann Whitney test	3-4	BIHi001	10-12	47	-55.17	1.669	0.2102
				BIHi004	9-11	31	-52.48	1.656	
2 A	Input resistance	two-tailed Mann Whitney test	3-4	BIHi001	10-12	47	387.3	28.56	0.61
				BIHi004	9-11	31	363.7	35.21	
2 A	Membrane capacitance	two-tailed Mann Whitney test	8-9	BIHi001	10-12	98	30.76	1.4	0.0564
				BIHi004	9-11	82	28.02	1.588	
2 D	Kainic acid	two-tailed Mann Whitney test	2	BIHi001	10	30	10.83	1.328	0.0155
				BIHi004	10	30	10.09	2.671	
2 D	NMDA	two-tailed Mann Whitney test	2	BIHi001	10	30	0.7179	0.2829	0.3099
				BIHi004	10	30	0.9838	0.3229	
2 D	GABA	two-tailed Mann Whitney test	2	BIHi001	10	30	92.9	12.59	0.0696
				BIHi004	10	30	67.02	12.37	
3 C	EPSC amplitude	two-tailed Mann Whitney test	4	BIHi001	15-20	70	1.055	0.1575	0.6432
				BIHi004	15-20	70	1.048	0.1412	
3 D	Sucrose charge	two-tailed Mann Whitney test	6	BIHi001	7-9	51	51.36	9.768	0.4425
				BIHi004	7-9	45	66.69	11.32	
3 E	# SV in the RRP	two-tailed Mann Whitney test	6	BIHi001	7-10	49	617.18	96.51	0.0592
				BIHi004	7-10	44	869.3	113.2	
3 F	P_{VR}	two-tailed Mann Whitney test	6	BIHi001	7-10	51	14.23	2.14	0.2885
				BIHi004	7-10	45	11.08	2.14	
3 G	mEPSC freq.	two-tailed Mann Whitney test	6	BIHi001	7-12	62	2.161	0.4345	0.4130

				BIHi004	7-12	63	1.782	0.3919	
3 H	mEPSC ampl.	two-tailed Mann Whitney test	6	BIHi001	7-12	62	39.71	2.448	0.0024
				BIHi004	7-12	63	30.35	2.778	
3 H	mEPSC charge	two-tailed Mann Whitney test	6	BIHi001	7-12	62	87.68	5.337	0.0607
				BIHi004	7-12	63	78.36	5.818	
3 I	Spont. Release rate	two-tailed Mann Whitney test	6	BIHi001	7-10	49	0.00814	0.001485	0.0615
				BIHi004	5-8	37	0.005834	0.002335	
3 J	PPR	two-tailed Mann Whitney test	4	BIHi001	10-15	50	0.7073	0.05868	0.1495
				BIHi004	10-15	51	0.7579	0.0433	
4 A	AMPA ratio	two-tailed Mann Whitney test	3	BIHi001	9-10	29	0.002097	0.002097	0.1209
				BIHi004	9-10	29	0.01326	0.006795	
4 B	NMDA ratio	two-tailed Mann Whitney test	3	BIHi001	9-10	29	1.018	0.04115	0.0529
				BIHi004	9-10	29	0.9554	0.07859	
4 B	Baclofen	two-tailed Mann Whitney test	3	BIHi001	9-10	29	0.3107	0.0418	0.1516
				BIHi004	10	30	0.2332	0.04206	
4 C	DCG IV	two-tailed Mann Whitney test	2-3	BIHi001	9-10	19	0.908	0.05378	0.2091
				BIHi004	10	30	0.8077	0.05082	
4 D	L-AP4	two-tailed Mann Whitney test	3	BIHi001	9-10	29	0.9779	0.03322	0.2127
				BIHi004	10	30	0.878	0.03775	
5 B	EPSC amplitude	two-tailed Mann Whitney test	2	BIHi001	10-15	24	0.8892	0.2453	<0.0001
				BIHi004	9-10	19	0	0	
5 C	Sucrose charge	two-tailed Mann Whitney test	2	BIHi001	6-9	15	0.05844	0.01996	<0.0001
				BIHi004	9-10	19	0	0	
